# Advancing Structural Health Monitoring: Accurate PCB Design for IoT-Based Real-Time Damage Detection with Digital Twin Integration

**DOI:** 10.3390/s26051672

**Published:** 2026-03-06

**Authors:** S. Adib, G. Ewart, V. Vinogradov, P. D. Gosling

**Affiliations:** School of Engineering, Newcastle University, Newcastle upon Tyne NE1 7RU, UK; graham.ewart@newcastle.ac.uk (G.E.); vladimir.vinogradov@newcastle.ac.uk (V.V.); peter.gosling@newcastle.ac.uk (P.D.G.)

**Keywords:** digital twin (DT), Internet of Things (IoT), Printed Circuit Board (PCB), proactive maintenance, structural health monitoring (SHM)

## Abstract

This paper introduces a cost-effective customised Printed Circuit Board (PCB) designed to establish an accurate Internet of Things (IoT) platform integrated with established Digital Twin (DT) models for advanced structural monitoring. The study focuses on developing a low-cost, precise PCB to synchronise real-time data between physical structures and their DT counterparts. The methodology includes a robust communication architecture utilising MQTT protocols, facilitating reliable data transmission and efficient integration with MATLAB for processing. Validation tests demonstrate high accuracy in data capture, with less than 1% deviation from conventional systems across multiple structural damage scenarios. This research highlights the potential of cost-effective PCB solutions for enhancing SHM and developing more resilient, proactive infrastructure management strategies.

## 1. Introduction

The integration of IoT technologies with DT models represents a new frontier in real-time monitoring and damage identification [1,2]. This combination provides engineers and infrastructure managers with a dynamic, virtual representation of physical assets that is continuously updated with real-time data. DTs enable detailed simulation, analysis, and prediction of structural behaviour under various conditions, significantly enhancing the ability to identify potential damage before it becomes critical [3]. Recent reviews have explored the evolution of digital twin systems for SHM, providing a roadmap for integrating simulation, sensing, and data-driven decision-making in civil infrastructure [4]. The integration of IoT with DTs offers comprehensive visibility into the operational status and health of infrastructure. By feeding real-time data from IoT sensors directly into DT models, a live, evolving simulation of the physical structure is maintained. This allows for the monitoring of structural changes as they happen, facilitating immediate responses to any detected anomalies [5]. For instance, if sensors detect an unexpected shift in a building’s foundation, the DT can simulate the implications of this shift on the building’s overall structural integrity, enabling rapid assessment and decision-making. A detailed review of DT technologies highlights infrastructure provenance, simulation strategies, and integration challenges across built-asset lifecycles [6]. Moreover, this integration provides a nuanced understanding of how structures degrade over time. Traditional SHM methods rely on periodic data and static models, which may not capture the full picture of a structure’s condition. In contrast, a DT that continuously integrates real-time data delivers a more accurate and up-to-date representation of structural health, accounting for deterioration, environmental impacts, and usage patterns. This dynamic model becomes a key tool for lifecycle management, extending the usable life of infrastructure by ensuring that maintenance and repairs are conducted proactively based on the most current data available.

Sensor technology is a vital component of the IoT ecosystem [6,7,8]. Recent advancements in sensor technology have led to a rapidly developing ecosystem [9,10] where sensors serve as primary data collection points, converting physical phenomena into digital data. These sensors range from simple temperature sensors to sophisticated imaging devices capable of real-time structural integrity assessments [11,12]. The wide variety and specificity of available sensors enable a customised approach to monitoring different aspects of a structure’s health, ensuring that the collected data is both relevant and actionable. Data processing and analytics play a crucial role in transforming raw data into meaningful insights. With the advent of edge computing, much of this processing can now occur on or near the device itself, reducing latency and relieving network bandwidth pressure [9,13]. Recent implementations have demonstrated low-cost, low-power edge computing systems specifically tailored for SHM in IoT environments [14]. This is especially important for applications requiring real-time analysis and response, such as dynamic load adjustments on bridges or immediate alerts for potential structural failures. Additionally, advanced algorithms and machine learning models can be applied to this data to predict future trends, identify anomalies, and suggest preventative or corrective actions. However, to fully harness their potential in SHM, recent developments must be considered, including state-of-the-art SHM systems for bridges, strategies for missing measurement data recovery, and the use of intelligent algorithms such as vibration-based damage identification under varying environmental conditions. Furthermore, emerging techniques such as data augmentation and adaptive optimisation neural networks have demonstrated enhanced accuracy in damage identification, particularly in complex systems such as steel bridges.

Additionally, using DTs alongside IoT technologies enables the simulation of various damage scenarios and their potential impact on the physical structure. This capability is particularly valuable for training and preparedness, allowing engineering teams to simulate responses to hypothetical damage scenarios, thereby enhancing the resilience of infrastructure to real-world challenges. Combining IoT and DTs for instantaneous monitoring and damage identification marks a significant leap forward in SHM [15,16,17]. This method enhances the precision and promptness of damage detection and offers a robust platform for predictive maintenance, operational refinement, and risk management. As this technology progresses, it is anticipated to become essential in safeguarding the safety, dependability, and durability of critical infrastructure globally. This paper introduces part of the author’s PhD work, which focuses on developing a customised, cost-effective PCB board to establish an accurate IoT platform for real-time communication between the physical structure and its digital twin during online monitoring and for damage identification as soon as it appears.

This paper defines the IoT as a network of interconnected physical devices equipped with sensors and communication protocols that enable them to collect, exchange, and transmit data in real time. A DT is understood as a virtual representation of a physical asset that continuously updates based on real-time data inputs, enabling simulation, prediction, and analysis of the asset’s performance and condition across its lifecycle.

## 2. Hardware Description

The customised PCB described in this paper is designed to facilitate real-time SHM by integrating advanced IoT technology with a reliable hardware framework. The PCB incorporates an ESP32 microcontroller, chosen for its robust computing capabilities, comprehensive wireless communication support, and ease of integration with peripheral modules. The board includes several key components that collectively enable precise and efficient data acquisition, transmission, and processing:

### 2.1. Component Layout

**Figure 1:**
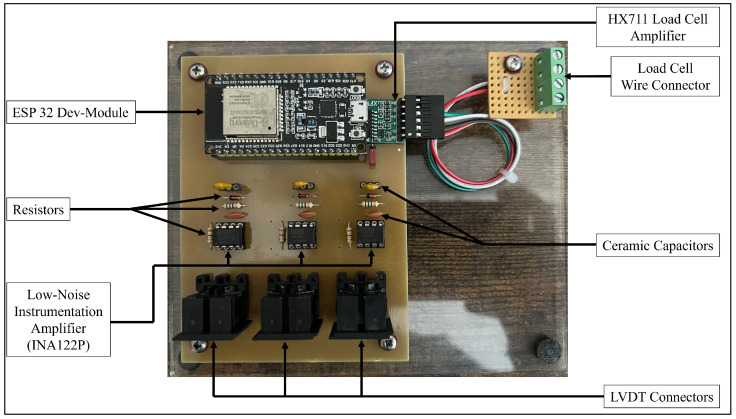
Updated PCB Board Component Layout.

The PCB is built on a standard FR-4 substrate, selected for its excellent electrical insulation and mechanical strength. The compact layout (Figure 1) includes resistors, capacitors, a bridge rectifier, and optocouplers. The bridge rectifier ensures power supply stability, while optocouplers provide electrical isolation, reducing noise and improving signal integrity. These components, while not visible in earlier images, are explicitly labelled in the updated layout and included in the bill of materials.

### 2.2. Circuit Design

**Figure 2:**
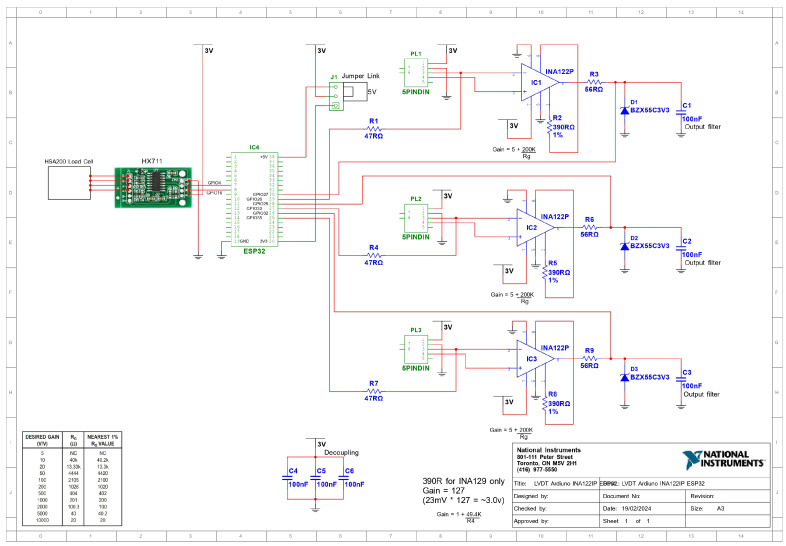
Detailed Circuit Diagram showing connections and components.

The circuit design (Figure 2) is optimised for noise immunity and precision. Low-noise instrumentation amplifiers ensure high signal integrity, while decoupling capacitors stabilise the voltage supply. Strategic placement of components minimises interference and enhances overall performance.

### 2.3. Sensor Integration and Connectivity

The PCB interfaces with a load cell via the HX711 ADC and with LVDT sensors for accurate displacement and load measurements. MQTT protocols are used for real-time data streaming to both a cloud server and MATLAB, ensuring seamless integration with the DT framework. Implementing PCA and autoencoder algorithms on low-power STM32 microcontrollers, this work demonstrates five-order reduction in bandwidth usage while retaining detection accuracy in a field-tested bridge SHM deployment [18].

### 2.4. Power Supply

The board is powered via USB, simplifying power management. While USB power may introduce noise in some scenarios, this has been mitigated using additional voltage regulators and filtering components. For applications requiring higher precision, a dedicated 5V power source can be incorporated.

### 2.5. Mechanical and Manufacturability Considerations

The PCB is designed for compact enclosures, with careful attention to avoiding mechanical and electrical interference. Standardised component packages ensure ease of manufacturability and facilitate quality assurance during production.

### 2.6. Key Advantages

- **Cost-Efficiency:** The PCB utilises affordable, widely available components, making it an economical choice for high-precision SHM applications. As summarised in Table 1, the total component cost remains under £150. In comparison, commercial alternatives such as National Instruments (NI) data acquisition systems with similar functionality typically cost over £3000. Even when accounting for labour costs associated with assembly and calibration—estimated at approximately £200—the overall expense remains considerably lower, offering a cost-effective solution for scalable deployment in real-time monitoring systems.
- **Customisation:** The open hardware design allows for modifications based on specific requirements, enhancing its versatility.
- **Ease of Use:** The integration with platforms like Arduino IDE and MATLAB simplifies programming, calibration, and data analysis.
- **Scalability:** The modular design supports a wide range of SHM scenarios, from small-scale experiments to large-scale infrastructure monitoring.
- **Real-Time Monitoring:** The robust design ensures reliable real-time monitoring, enabling proactive maintenance and rapid response to structural anomalies.

The proposed PCB offers an innovative solution for researchers and engineers, balancing affordability, customisability, and performance. Its implementation demonstrates the feasibility of integrating IoT technology with SHM frameworks, paving the way for advancements in infrastructure resilience and longevity.

## 3. Bill of Materials Summary

Table 1 presents a summary of all electronic components used in the proposed system, including their designators, quantities, unit costs, total costs, and sources of procurement.

**Table 1:** Summary of components and their costs.

Designator	Component	Number	Cost Per Unit (£)	Total Cost (£)	Source of Materials
ESP32-DevKitC	ESP32 Dev-module	1	10.99	10.99	AZ-Delivery (https://www.az-delivery.uk/products/esp32-developmentboard, accessed on 30 November 2024)
HX711	HX711 Load Cell Amplifier	1	1.17	1.17	AEDIKO (https://www.amazon.co.uk/)
C1–C6	Ceramic Capacitor	6	0.03	0.18	BOJACK (https://www.amazon.co.uk/)
R1–R9	Resistor	9	0.05	0.45	BOJACK (https://www.amazon.co.uk/)
INA122P	Low-noise instrumentation amplifier	3	4.63	13.89	Mouser (https://www.mouser.co.uk/)
DIN-5	Breadboard-friendly MIDI Jack (5-pin DIN)	3	1.50	4.50	Texas Instruments (https://www.ti.com/)
**Total**					**£31.18**

## 4. Build Instructions

### 4.1. Step-by-Step Construction Instructions

Begin by gathering all necessary components listed in the Bill of Materials, such as the ESP32 Dev-module, HX711 Load Cell Amplifier, ceramic capacitors (C1–C6), resistors (R1–R9), INA122P low-noise instrumentation amplifiers, and a breadboard-friendly MIDI Jack (5-pin DIN). Ensure the availability of essential tools including a soldering iron, solder, wire cutters, and a multimeter. The PCB design and layout should be created using software like Eagle or KiCad, ensuring the layout accommodates all components as per the schematic. Once the design is complete, print and etch it onto a standard FR-4 PCB, or alternatively, utilise a PCB manufacturing service. Drill any necessary holes for through-hole components to ensure proper assembly.

Place the components carefully on the PCB. Start with the ESP32 Dev-module, positioning it in its designated area. Place the HX711 Load Cell Amplifier near the load cell connector. Solder the ceramic capacitors (C1–C6), resistors (R1–R9), and INA122P amplifiers in their respective positions, following the schematic closely. Attach the breadboard-friendly MIDI Jacks at their assigned locations to facilitate sensor connections. Solder all components to the PCB, taking care to ensure strong connections and avoid solder bridges. Connect the LVDT sensors and load cell to their corresponding connectors on the PCB. Power and data communication can be established through the micro USB port on the ESP32 Dev-module.

Next, connect the ESP32 to your computer using a micro USB cable. Open the Arduino IDE and install the required libraries for the ESP32 and HX711. Upload the provided code to the ESP32, ensuring the code includes the necessary MQTT server details for cloud communication. Integrate the system with MATLAB by installing MATLAB software and the Arduino Explorer Toolbox. Use the Arduino Explorer app to establish a connection between MATLAB and the ESP32. Configure MATLAB to read sensor data from the ESP32, calibrate the data, and perform real-time analysis.

**Figure 3:**
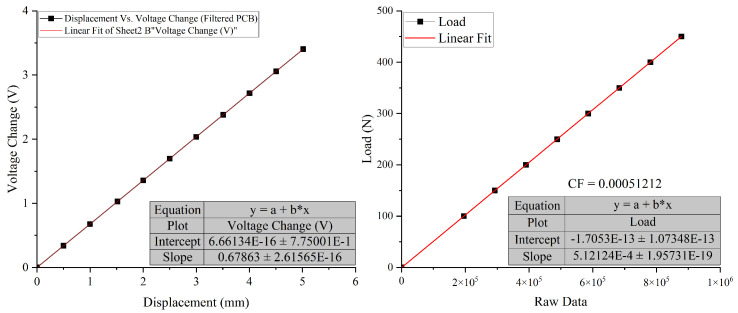
LVDT and Load Cell Calibration Factor.

To test and calibrate the system, power on the PCB and connect it to the cloud server. Verify data transmission from the sensors to the cloud and MATLAB. Calibrate the sensors using known loads and displacements, making adjustments to the calibration factors in the MATLAB script as necessary. Figure 3 presents the calibration graphs for the LVDT and Load Cell sensors. Beyond laboratory calibration, the system is designed for real-world structural monitoring and is capable of measuring load and displacement in field applications, including temperature-induced bearing displacement. This makes it suitable for integration into advanced monitoring frameworks, such as those employing data-driven models such as DCNN-LSTM to capture non-linear structural responses under varying environmental conditions.

### 4.2. Design Decisions and Alternatives

The ESP32 Dev-module was chosen for its robust wireless capabilities and ease of integration with sensors and cloud services. While alternatives such as an Arduino with an external Wi-Fi module exist, they could complicate the setup. The HX711 Load Cell Amplifier was selected for its precision and cost-effectiveness; higher-end amplifiers may offer better performance but at increased cost. The PCB design prioritised a compact and stable layout using standard FR-4 material for its balance of cost and durability, though flexible PCBs could be considered for dynamic applications.

## 5. Operation Instructions

### 5.1. Step-by-Step Operational Instructions

To begin, ensure the ESP32 board is securely connected to the PCB and all sensors, including LVDTs and load cells, are properly attached to their respective ports. Inspect all connections to verify that the wiring is secure and properly aligned to avoid signal interference or connection issues. Connect the power supply to the ESP32 through the micro USB port, verifying that the connection is stable. Power the system by plugging the USB cable from the ESP32 board into a computer or power adapter. If the ESP32 board is not recognised by the computer, download and install the necessary drivers from the manufacturer’s website. Ensure that the board is detected without errors, as proper recognition is critical for reliable communication and data transfer.

Using the Arduino IDE, upload the provided code to the ESP32 board. This code establishes the connection to the cloud server, configures the sensors, and sets data acquisition parameters. Double-check the code for errors and confirm the use of the correct board settings in the IDE. Configure the ESP32 to connect to Wi-Fi by entering the credentials in the code prior to uploading. Once uploaded, the ESP32 should automatically establish a connection to the MQTT broker. Use the serial monitor in the Arduino IDE to confirm that the connection to the broker is successful and that the sensors are publishing data as expected.

**Figure 4:**
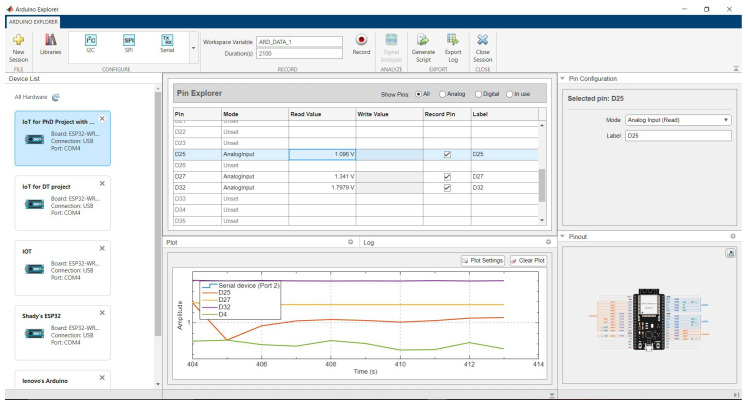
Arduino Explorer app interface used for data stream monitoring.

Sensor calibration is critical for accurate measurements. For LVDT sensors, compare the readings with precision micrometer measurements and adjust calibration constants in the code as necessary. For the load cell, use the dead weight method by applying known weights and recording the corresponding outputs to establish a calibration curve. Include detailed documentation of the calibration process, ensuring reproducibility for future users. After calibration, initiate the data acquisition process. The ESP32 will read sensor data and publish it to the MQTT broker at defined intervals. Although CloudMQTT was used for initial testing due to its ease of setup and accessibility, it is important to note that the system is fully compatible with other MQTT brokers such as Mosquitto, HiveMQ, or Eclipse EMQX. This ensures long-term flexibility, stability, and adaptability of the system for deployment in various environments. Utilise MATLAB’s Arduino Explorer app to capture and analyse the data in real time, converting raw readings into meaningful displacement and load measurements. The interface of the Arduino Explorer app for data stream monitoring is shown in Figure 4.

### 5.2. Visual Instructions

**Figure 5:**
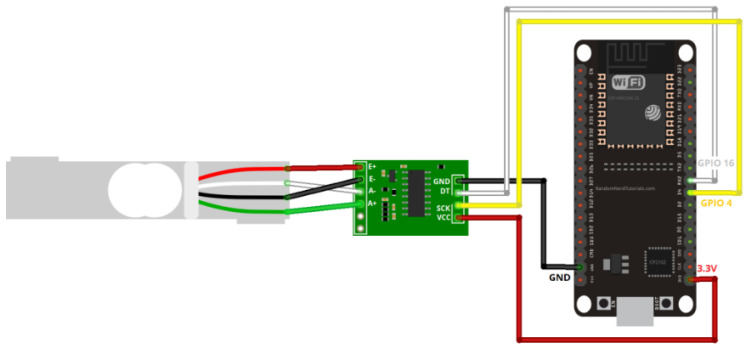
Wiring diagram for load cell, HX711 amplifier, and ESP32 board.

**Figure 6:**
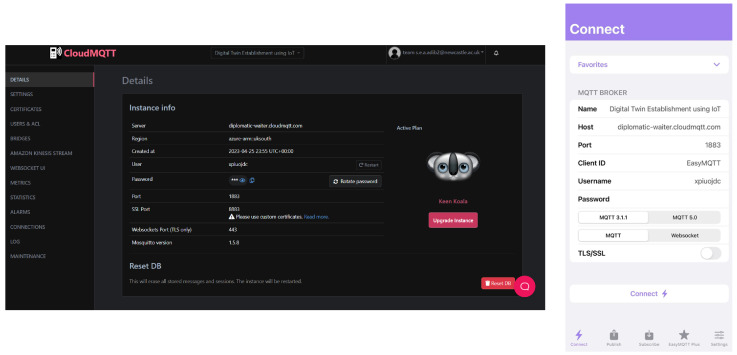
MQTT server setup and configuration.

Figure 5 illustrates the wiring setup between the load cell, HX711 amplifier, and ESP32 board. Clearly label each component to ensure proper assembly and avoid misconnection issues. This setup ensures accurate load measurements. Figure 6 shows the configuration for MQTT broker applications, facilitating data transmission to the cloud. Both figures are updated to include a high-level schematic and component labels for better clarity.

### 5.3. Safety Considerations

Ensure all connections are secure to prevent short circuits or loose wiring, which could lead to damage or pose a fire hazard. Handle the PCB and electronic components carefully to avoid static discharge; use anti-static wrist straps if available. Verify that all connections are properly insulated to reduce the risk of accidental shorting. Operate the system in a dry and dust-free environment to minimise the risk of electrical shock or component failure due to moisture or contamination. Always disconnect the power supply before making hardware adjustments to ensure user safety and prevent potential damage to the components. Test the system in a controlled environment to confirm proper functionality before deploying it in operational settings.

## 6. Validation and Characterisation

To validate the developed PCB board and characterise its performance, its operation was demonstrated in a specific scientific application, with an assessment of its capabilities and limitations.

### 6.1. Demonstration of Operation

The developed PCB board was evaluated for its capability to achieve real-time synchronisation between the physical structure and its DT. The focus was on assessing the accuracy of the PCB board for establishment of an accurate IoT platform for identifying the structure’s status across various scenarios, in comparison with the experimental outcomes obtained using the National Instrument (NI) system and the DT predictions generated by the developed method. The objective was to ascertain whether the IoT system could match or surpass the benchmark set by the NI system in capturing structural responses under different test conditions and hence establishment of accuate DT models. This case study shows how low-cost wireless accelerometer networks and ML can support digital-twin based SHM on an active railway bridge with automated anomaly detection over two years [5].

**Figure 7:**
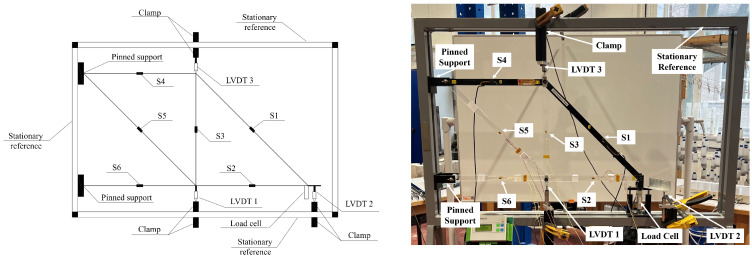
Graphical and real experimental setup.

The experimental investigation was conducted at Newcastle University’s Heavy Structures Lab to validate the IoT-based customised PCB board. The primary objective was to assess the structure’s behaviour by measuring displacements in a 2D Acrylic truss under load, which ranged from 50 N to 300 N. The truss was originally manufactured by P.A. Hilton Ltd., Hampshire, UK, a renowned company with a long history of providing high-quality educational and research equipment in structural engineering. The specific model used, HST17S, is part of their Forces in a Truss (Resolution) Experiment Software, known for its precision and reliability in educational settings. The truss elements are made of Clear Cast Perspex Acrylic Sheet, known for its excellent clarity and durability. For this project, modifications were made to introduce artificial damage to the manufactured elements by reducing the cross-sectional area of the truss elements to different lengths, thereby creating varying levels of damage. The experiment involved replacing certain original truss elements with these modified elements to test various damage scenarios. The setup, illustrated in Figure 7, included three LVDTs and six strain gauges, strategically placed to measure vertical displacements and axial strains, respectively. The load was applied according to a predefined load-time profile, repeated four times to ensure stability. Data from the LVDTs, strain gauges, and load cell were recorded using a NI system to compare and validate the results with the IoT system.

### 6.2. Performance Characterisation

#### 6.2.1. Summary of Capabilities and Limitations

- **Accuracy:** The PCB board demonstrated high accuracy in load and displacement measurements, with percentage differences between the IoT system and the NI system remaining below 1% across various damage levels.
- **Real-Time Monitoring:** The PCB board effectively monitored structural integrity in real-time, providing comparable data to the NI system.
- **Robustness:** The system maintained accuracy and reliability even as damage levels increased up to 15.68%.
- **Real-Time Monitoring:** The PCB board effectively monitored structural integrity in real-time, providing comparable data to the NI system. A secure edge-computing reference architecture deployed on a real bridge system (“Living Bridge”) offers extensive benchmarking of ML model performance on ARM/Linux devices for SHM applications [19].

#### 6.2.2. Comparative Analysis of PCB Board Performance in Test 1

##### Experimental Setup

Test 1 was designed to verify the functionality of the developed PCB board by introducing damage at a single location, Element E3 (corresponding to sensor S3), on the truss with varying levels of severity. The damage was physically simulated by progressively reducing the cross-sectional area of Element E3 through controlled drilling, representing a quantifiable reduction in stiffness. Five levels of damage were introduced, as summarised in Table 2, each corresponding to a specific percentage of material loss. The location of the damage was chosen to ensure consistent sensor response, while allowing for the evaluation of the system’s sensitivity to varying damage severities in a controlled and repeatable manner.

##### Summary Table

The comparison results between NI and IoT systems for Test 1, where a single damage location was introduced to the truss, are shown in Table 2. The table presents a comparison of load and displacement measurements between the two systems across different cases with varying levels of damage.

##### Overlay Graphs

**Figure 8:**
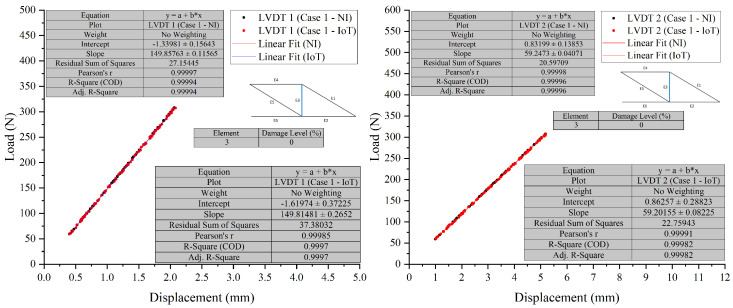
Case 1 comparison of LVDT 1 and LVDT 2 response: NI vs. IoT.

**Figure 9:**
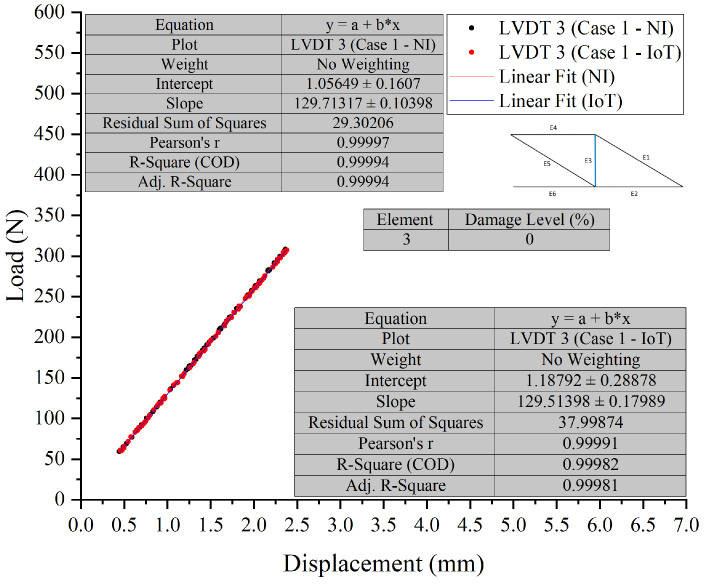
Case 1 comparison of LVDT 3 response: NI vs. IoT.

**Figure 10:**
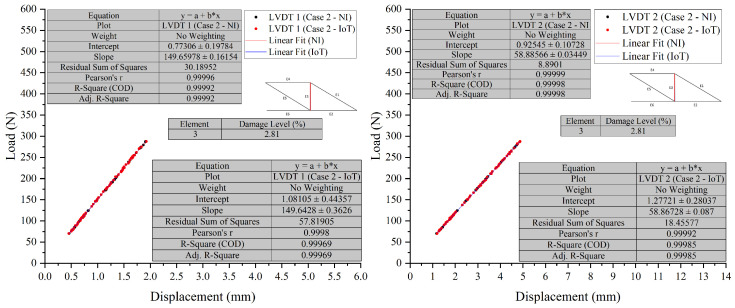
Case 2 comparison of LVDT 1 and LVDT 2 response: NI vs. IoT.

**Figure 11:**
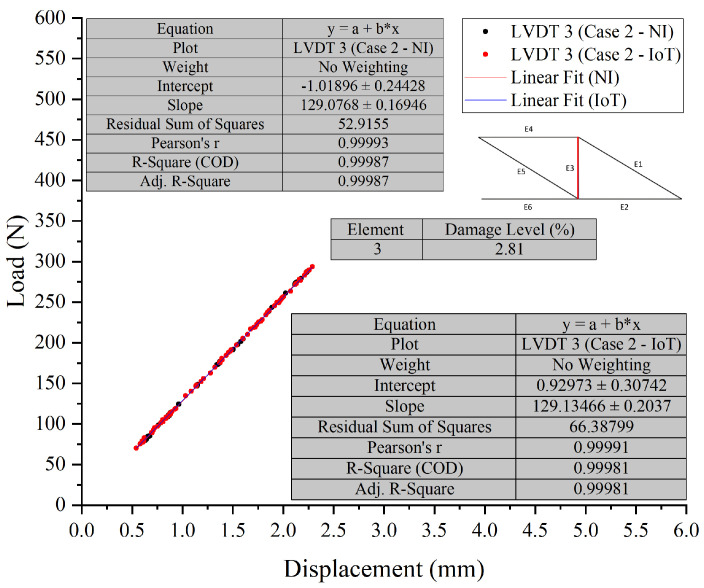
Case 2 comparison of LVDT 3 response: NI vs. IoT.

To provide clear evidence and validation of the system’s capabilities, overlay graphs compare the NI system and the IoT system responses under different damage conditions, as shown in Figure 8, Figure 9, Figure 10 and Figure 11. These figures illustrate the system’s performance under varying damage scenarios. The overlay graphs show a close match between the load–displacement responses recorded by the NI and IoT systems, confirming the reliability of the IoT system in replicating the traditional system’s performance.

##### Differential Analysis and Discussion

The experimental results, illustrated in Figure 8, Figure 9, Figure 10 and Figure 11 and summarised in Table 2, demonstrate the PCB board’s capability to monitor the truss’s structural integrity accurately. The experimental results, shown through superimposed graphs and a summary table, demonstrate the PCB’s capability to monitor the truss’s structural integrity accurately. The summary table provides a comparison of load and displacement from both the NI and IoT systems. The analysis, conducted under different damage conditions, shows a consistent match, with slight measurement differences between the systems. This accuracy is retained across various damage levels, indicating the PCB’s reliability. The data, which includes load and displacement, shows the IoT system generally reports marginally lower values than the NI system. However, the small variance seen, even with greater damage, suggests that the IoT system’s performance is comparable to that of the NI system. The graphs support this, showing a close correlation between the load-displacement lines. The combination of graphical and tabular data shows that the PCB, enhanced with IoT, matches the SHM process provided by the NI system. The fusion of traditional accuracy with the advanced features of IoT marks progress, indicating improved precision in real-time structural monitoring.

#### 6.2.3. Comparative Analysis of PCB Board Performance in Test 2

##### Experimental Setup

Test 2 involved exposing the truss to multiple damage locations to assess the developed board’s performance in more complex scenarios.

##### Overlay Graphs

In Test 2, ten cases were tested. Case 9 and Case 10, were chosen to assess the developed PCB board’s ability to detect the location and severity of damage in scenarios where multiple damage locations were introduced to the truss.

**Figure 12:**
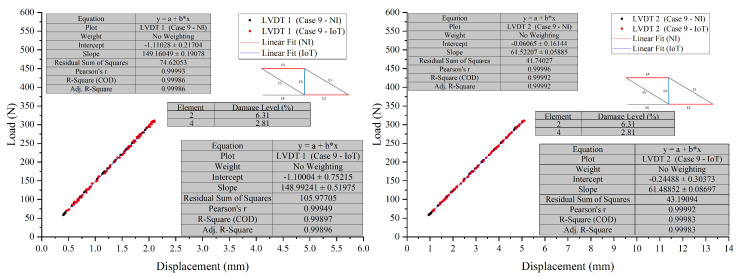
Case 9 comparison of LVDT 1 and LVDT 2 response: NI vs. IoT.

**Figure 13:**
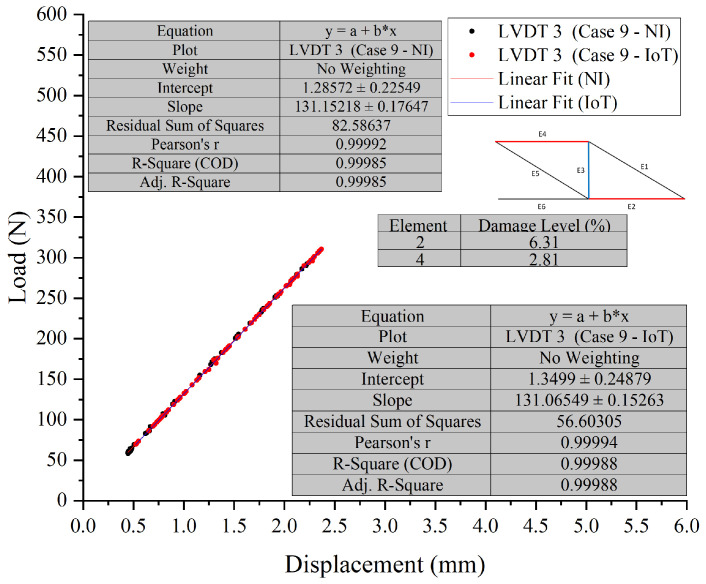
Case 9 comparison of LVDT 3 response: NI vs. IoT.

**Figure 14:**
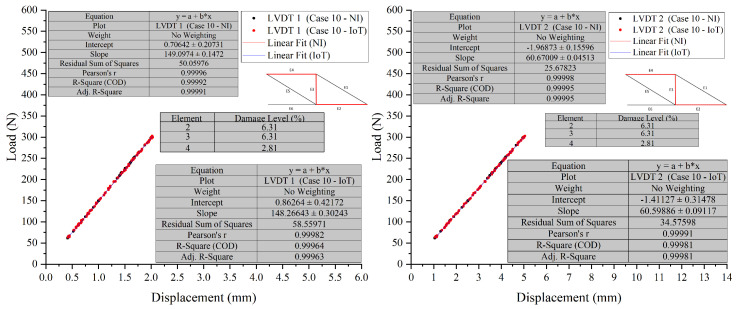
Case 10 comparison of LVDT 1 and LVDT 2 response: NI vs. IoT.

**Figure 15:**
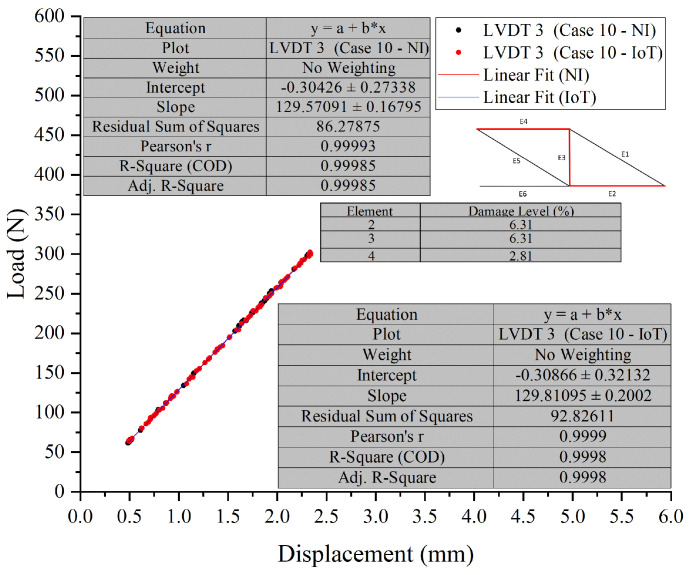
Case 10 comparison of LVDT 3 response: NI vs. IoT.

The visual representation of Case 9, see Figure 12 and Figure 13, indicates that the data points overlap closely, signifying that both the NI and IoT systems provide similar readings for the displacement under load for the given elements in the structure. This indicates a high level of agreement between the measurements of the two systems. It can be concluded that the IoT-based system’s performance is comparable to that of the NI system in terms of accuracy for the presented case, Case 10, see Figure 14 and Figure 15, shown in the figures below. Both systems were able to collect load data across a range of displacements with a high degree of overlap, suggesting that the IoT system is a viable alternative to the NI system for this application.

##### Comparative Analysis and Discussion

In Test 2, a detailed comparison was made between NI and IoT systems, focusing on their performance in scenarios with multiple damage locations on a truss structure. The results showed only minor differences in load and displacement measurements between the two systems, suggesting comparable accuracy. Despite the complexities of multiple damage locations, the IoT system’s readings closely aligned with those from the NI system, demonstrating its reliability and precision. The integration of IoT technology was found to be compatible with the LVDTs’ measurement capabilities. Across various cases and sensors, the data showed consistency, indicating a well-calibrated system providing accurate and consistent measurements. While the IoT system occasionally reported slightly lower displacement values, the discrepancies were not substantial enough to compromise the reliability of the results. The effectiveness of the PCB in detecting damage location and severity was confirmed by the high level of agreement in measurements between the two systems, with the figures showing closely aligned data points. This reinforces the viability of the IoT system as a reliable alternative to the NI system for such tests.

### 6.3. Assessment of Online Damage Detection and Severity Prediction Using the Developed PCB Board

**Figure 16:**
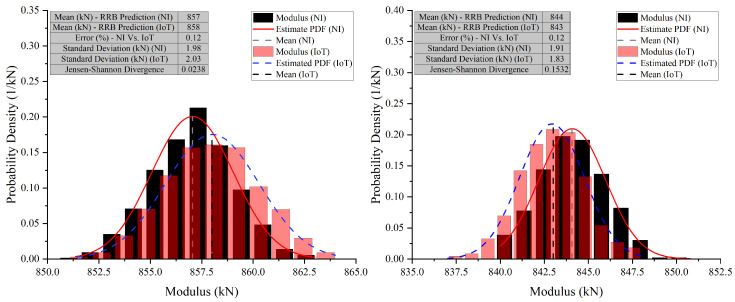
Comparative analysis of modulus predictions by NI and IoT systems—Case 1 and 2 (Test 1).

The DT model prediction was evaluated using the IoT system against the NI system, as shown in Figure 16, where overlapping between the two PDFs, derived from Case 1 and Case 2 from Test 1, were plotted. These PDFs were formulated from the data points of recorded displacement from the three LVDTs with the loads spanning from 50 to an average of 300 N. Importantly, the selection of these data points adhered to the data selection criteria ensuring that the analysis was grounded in a rigorous and consistent methodological approach. The mean and standard deviation for each case are presented in the descriptive statistics table shown in the graph. Additionally, Jensen-Shannon Divergence (JSD) is calculated to provide a measure of similarity between the two PDFs. JSD values close to 0 denote a strong similarity, while values nearing 1 indicate substantial dissimilarities. It’s important to note that the x-axis in these figures has a very narrow range. This detail highlights the close agreement between the NI and IoT systems, making the overlap of the PDFs more visually compelling.

(1) $$JSD\left(P∥Q\right)=\frac{1}{2}\sum_{x}P\left(x\right)\log\left(\frac{P(x)}{M(x)}\right)+\frac{1}{2}\sum_{x}Q\left(x\right)\log\left(\frac{Q(x)}{M(x)}\right)$$

where P(x) and Q(x) are the probabilities of *x* in distributions *P* and *Q*, respectively and $M\left(x\right)=\frac{1}{2}\left(P\left(x\right)+Q\left(x\right)\right)$ is the average probability of *x* in *P* and *Q*.

**Figure 17:**
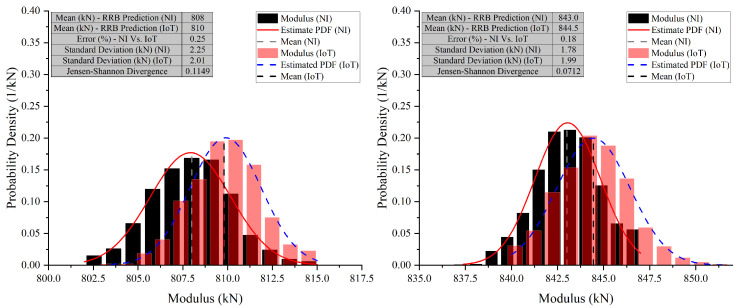
Comparative analysis of modulus predictions by NI and IoT systems—Case 9 (E2) and (E4) (Test 2).

**Figure 18:**
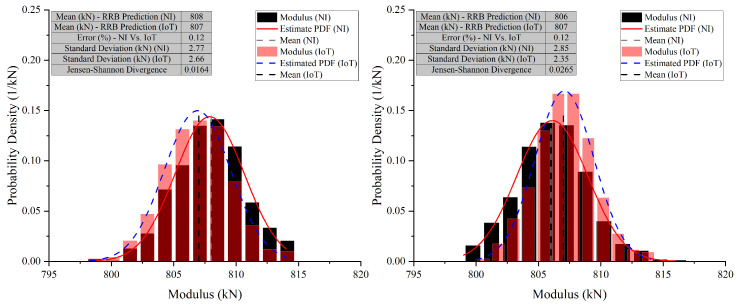
Comparative analysis of modulus predictions by NI and IoT systems—Case 10 (E2) and (E3) (Test 2).

**Figure 19:**
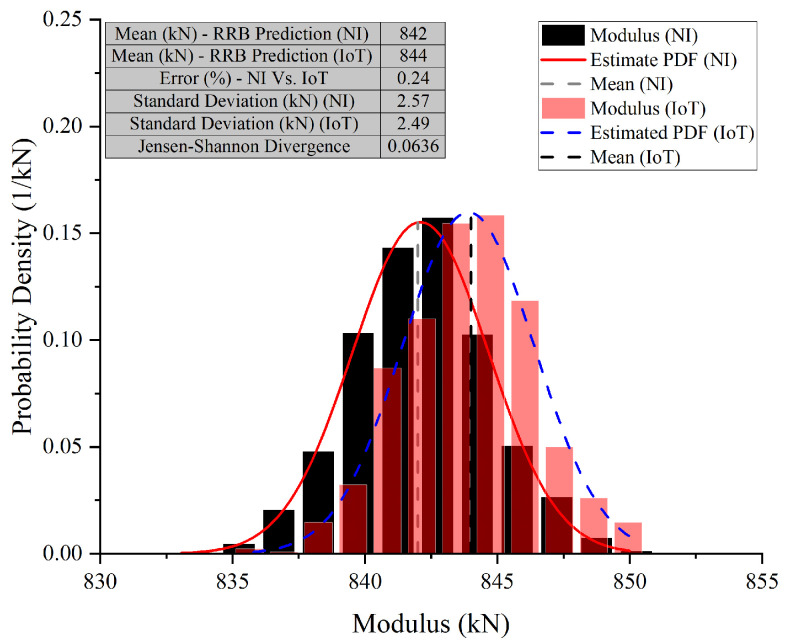
Comparative analysis of modulus predictions by NI and IoT systems—Case 10 (E4) (Test 2).

In Test 2, Case 9 and Case 10 were selected to compare the developed PCB board against the NI system (Figure 17, Figure 18 and Figure 19). In Case 9 (E2), NI’s mean modulus prediction is 808 kN, while IoT’s is slightly higher at 810 kN. The standard deviations are 2.25 kN for NI and 2.01 kN for IoT, showing IoT’s predictions are more consistent. The JSD value of 0.1149 indicates similar probability distributions with minor differences. In Case 9 (E4), NI’s mean prediction is 843 kN, slightly lower than IoT’s 844.5 kN. The standard deviations are 1.78 kN for NI and 1.99 kN for IoT, indicating more consistent predictions from NI. The JSD value of 0.0712 shows a closer alignment between the NI and IoT distributions compared to Case 9 (E2). The narrow x-axis range in these graphs emphasizes the small differences and the close alignment between the predictions of the NI and IoT systems.

In both cases, the IoT system tends to predict a slightly higher modulus value on average than the NI system. However, the spread of predictions, as indicated by the standard deviation, varies between the cases, with IoT being more consistent in Case 9 (E2) and NI in Case 9 (E4). The JSD values suggest that the prediction distributions have some differences, with Case 9 (E4) showing a higher degree of similarity between NI and IoT predictions than Case 9 (E2).

In Case 10 (E2), the NI system predicted a mean modulus of 808 kN, while the IoT system predicted 807 kN. The standard deviations were close, with NI at 2.77 kN and IoT at 2.66 kN, indicating similar variability. The error between the NI and IoT systems was low at 0.12%, and the JSD was 0.0164, suggesting nearly identical probability distributions. In Case 10 (E3), the NI system’s mean modulus prediction was 806 kN, slightly lower than the IoT’s 807 kN. The standard deviation for NI was 2.85 kN, slightly higher than IoT’s 2.35 kN. The error remained low at 0.12%, showing strong alignment in predictions. The JSD increased slightly to 0.0265, still indicating high similarity between the probability distributions. In Case 10 (E4), the NI system predicted a mean modulus of 842 kN, while the IoT system predicted 844 kN. Standard deviations were close, with NI at 2.57 kN and IoT at 2.49 kN, suggesting comparable precision. The error percentage increased slightly to 0.24%, showing a small discrepancy between the systems. The JSD was 0.0636, indicating a slight difference in the distribution of predictions. The graphs for these cases also show a narrow x-axis range, underscoring the close agreement and making even minor differences more apparent.

### 6.4. Analysis of DT Performance Using IoT Technology

The analysis of the DT performance, using IoT technology, offers insights into its use in SHM. The experimental results, presented through graphs and tables, show that the developed PCB board is capable of accurately monitoring and assessing the structural integrity of the truss. Comparisons of load and displacement measurements from both NI systems and the new IoT systems demonstrate good consistency. This indicates that the PCB board is reliable in its measurement capabilities, and the IoT system’s performance is comparable to that of the NI system, suggesting a positive contribution to the SHM process. Further comparisons, especially in cases with multiple damage locations on a truss structure, confirm the IoT system’s accuracy is similar to the NI system. The minimal differences observed highlight the IoT system’s reliability. These results suggest that using cost-effective PCB board for establishing an accurate IoT platform does not compromise measurement accuracy, even in complex scenarios, making it a feasible option for integration into SHM practices.

The IoT effectiveness in capturing accurate data for identifying damage location and severity was examined in further cases, with overlaid graphs demonstrating the consistency between the NI and IoT systems’ displacement measurements under various loads. This consistency suggests that the IoT system is a viable alternative to the NI system for SHM applications. Additionally, the analysis of modulus predictions, indicated by the low JSD values, shows a close similarity between the probability distributions of NI and IoT modulus predictions. Despite minor differences in mean predictions and standard deviations, this points to both systems being well calibrated, with the IoT system showing slightly higher precision in some scenarios. The empirical data and comparative analysis support the effectiveness of using IoT technology in real-time structural analysis and monitoring. The integration of IoT with traditional accuracy introduces enhanced precision in SHM, demonstrating the potential of IoT technology not only to match the accuracy of traditional systems but also to improve the monitoring process. This research presents a positive perspective on the future of structural integrity assessment through the use of IoT technology.

## 7. Conclusions

This study introduces a customised, cost-effective PCB designed to enhance real-time SHM by integrating IoT technology with DT models. Through extensive validation and characterisation, the PCB demonstrated high accuracy in load and displacement measurements, achieving performance metrics comparable to the traditional NI system. Validation tests revealed that the PCB consistently maintained measurement accuracy with less than 1% deviation from the NI system across varying damage levels, underscoring its reliability for SHM applications. Rigorous comparative analyses further highlighted the system’s robustness and ability to handle complex structural scenarios involving multiple damage locations, with seamless integration into DT frameworks.

The integration of IoT technology enabled real-time data transfer and synchronisation, ensuring continuous updates between the physical structure and its DT. Analysis using Jensen-Shannon Divergence (JSD) confirmed a high degree of similarity between the probability distributions of modulus predictions obtained from the NI and IoT systems, reinforcing the accuracy and reliability of the proposed system. While minor discrepancies were observed, these did not significantly affect the system’s performance, validating the PCB’s potential as a cost-effective and reliable alternative to traditional SHM solutions. In addition to addressing cost and accessibility barriers, this study emphasises reproducibility by providing detailed build instructions and open access to design files, fostering broader adoption and future research. The inclusion of hardware validation against established systems and an exploration of its limitations ensures that the PCB can be effectively scaled for diverse SHM applications.

In conclusion, the developed PCB, integrated with IoT and DT technologies, represents a meaningful advancement in SHM. Its combination of cost-effectiveness, high accuracy, and real-time monitoring capabilities offers a robust and adaptable solution for proactive infrastructure maintenance and damage mitigation. A recent framework identifies both technical and governance challenges for seamless SHM infusion with BIM/DT models, crucial for scalable infrastructure deployment [20]. By bridging the gap between emerging IoT technologies and practical SHM applications, this research contributes to the development of more resilient and self-aware infrastructures, paving the way for advancements in digital monitoring and predictive maintenance systems.

## Figures and Tables

**Table 2:** Performance comparison of IoT and NI systems across different damage scenarios—Test 1.

Case	LVDT	Damage Level (%)	$P_{\mathrm{NI}}$ (N)	$P_{\mathrm{IoT}}$ (N)	ΔP (%)	$\delta_{\mathrm{NI}}$ (mm)	$\delta_{\mathrm{IoT}}$ (mm)	Δδ (%)
1	1	0	159.57	158.71	0.54	1.0697	1.0604	0.86
	2					2.6696	2.6660	0.13
	3					1.2174	1.2143	0.25
2	1	2.81	148.64	147.36	0.86	0.9866	0.9804	0.63
	2					2.5005	2.4805	0.80
	3					1.1521	1.1422	0.85
3	1	6.31	148.67	149.38	0.48	0.9996	1.0025	0.29
	2					2.5601	2.5752	0.58
	3					1.1673	1.1728	0.47
4	1	10.50	149.83	148.70	0.75	1.0080	1.0045	0.94
	2					2.5736	2.5618	0.46
	3					1.1666	1.1620	0.39
5	1	15.68	147.79	148.06	0.18	1.0189	1.0177	0.12
	2					2.5478	2.5576	0.38
	3					1.1803	1.1783	0.17

## Data Availability

The data presented in this study are available on request from the corresponding author. The data are not publicly available due to restrictions related to institutional laboratory data and ongoing research usage.
